# let-7b/g silencing activates AKT signaling to promote gastric carcinogenesis

**DOI:** 10.1186/s12967-014-0281-3

**Published:** 2014-10-05

**Authors:** Wei Kang, Joanna HM Tong, Raymond WM Lung, Yujuan Dong, Weiqin Yang, Yi Pan, Kin Mang Lau, Jun Yu, Alfred SL Cheng, Ka Fai To

**Affiliations:** Department of Anatomical and Cellular Pathology, State Key Laboratory in Oncology in South China, Prince of Wales Hospital, The Chinese University of Hong Kong, Hong Kong, SAR People’s Republic of China; Institute of Digestive Disease, Partner State Key Laboratory of Digestive Disease, The Chinese University of Hong Kong, Hong Kong, SAR People’s Republic of China; Li Ka Shing Institute of Health Science, Sir Y.K. Pao Cancer Center, The Chinese University of Hong Kong, Hong Kong, SAR People’s Republic of China; Shenzhen Research Institute, The Chinese University of Hong Kong, Shenzhen, People’s Republic of China; Department of Medicine and Therapeutics, The Chinese University of Hong Kong, Hong Kong, People’s Republic of China; School of Biomedical Sciences, The Chinese University of Hong Kong, Hong Kong, People’s Republic of China

**Keywords:** let-7b, let-7g, AKT2, Gastric cancer, Tumor suppressor

## Abstract

**Background:**

Aberrant AKT activation contributes to gastric cancer cell survival and chemotherapy resistance, however its regulation is poorly understood. microRNAs have been established to be important regulators in gastric carcinogenesis. Here, we showed the functional role and putative target of let-7b and let-7g (let-7b/g) in gastric carcinogenesis.

**Methods:**

The expression of let-7b/g in gastric cancer cell lines and primary tumors were evaluated by miRNA qRT-PCR. The putative target gene of let-7b/g was explored by TargetScan followed by further validation. Functional analyses including MTT proliferation, monolayer colony formation, cell invasion assays and *in vivo* study were performed in both ectopic expression and knockdown approaches.

**Results:**

let-7b/g was found down-regulated in gastric cancer and its downregulation was associated with poor survival and correlated with lymph node metastasis. let-7b/g inhibited AKT2 expression by directly binding to its 3’UTR, reduced p-AKT (S473) activation and suppressed expression of the downstream effector pS6. AKT2 mRNA expression showed negative correlation with the expression of let-7b/g in primary tumors. Short interfering RNA (siRNA) mediated knockdown of AKT2 phenocopied the tumor-suppressive effects of let-7b/g. Moreover, AKT2 re-expression partly abrogated the growth-inhibitory effect of let-7b/g.

**Conclusion:**

In conclusion, our findings reveal decreased let-7b/g contributes to aberrant AKT activation in gastric tumorigenesis and provide a potential therapeutic strategy for gastric cancer.

**Electronic supplementary material:**

The online version of this article (doi:10.1186/s12967-014-0281-3) contains supplementary material, which is available to authorized users.

## Background

Gastric cancer is the fourth most common cancer worldwide, with an estimated one million new cases per year [1]. *H. pylori* is the most important risk factor in gastric tumorigenesis which induces gastric cancer potentially by chronic inflammation or through the action of *H. pylori* virulence factors such as CagA [2]. Approximately 95% of gastric cancer are adenocarcinomas by histological phenotype as intestinal type, diffuse type and mixed/unclassifiable according to Lauren’s classification [3]. Nowadays, several molecular classifications of gastric cancer have been proposed based on the analysis of whole-genome gene expression studies or deep sequencing studies [4]. Most gastric cancer patients are diagnosed at the advanced stage often accompanied with extensive invasion and lymphatic metastasis. Thus the investigations into the molecular mechanisms involving in gastric cancer progression become imperative and urgent for targeted therapy.

microRNAs (miRNAs) are a class of small non-protein-coding RNAs which have been identified as a new kind of gene expression regulators through binding to the 3′ untranslated regions (3′UTRs) of target mRNA, thus blocking mRNA translation or resulting in mRNA degradation [5,6]. Emerging evidence shows that miRNAs are abnormally expressed in cancer development and the deregulated miRNAs are associated with tumor initiation, promotion and progression by regulating target genes expression [7]. Some miRNAs such as miR-372, −544, −25, −373 show up-regulated expression and exert oncogenic property in gastric cancer [8-10]. On the contrary, miR-9, −202-3p, −7 and −206 are down-regulated and play tumor suppressor function in gastric carcinogenesis [11-14].

By using miRNA expression microarray, we have identified aberrantly expressed miRNAs in gastric cancer cell lines including let-7 family. Compared with normal gastric epithelium tissue, let-7 family was found to be down-regulated in all 9 gastric cancer cell lines (Additional file 1: Table S1), in which let-7a/b/f and let-7c/d/e/g/i were down-regulated for thousand and hundred times respectively. let-7 microRNA was first identified in gastric cancer due to its targeting on high mobility group A2 (HMGA2) [15], a nonhistone chromosomal protein that can modulate transcription by altering chromatin architecture. It has been reported that the expression of let-7 in gastric cancer correlated with *H. pylori* infection [16]. Hayashi reported that *H. pylori* CagA induced aberrant epigenetic silencing of let-7 expression [17]. let-7f, a member of let-7 family, is able to inhibit tumor invasion and metastasis by targeting MYH9 in human gastric cancer [18]. However, for let-7b and let-7g (let-7b/g), the possible role and putative target genes in gastric cancer cells are still not well elucidated and need investigation.

## Materials and methods

### Cell line and primary gastric tissues

The information of human gastric cancer cell lines (MKN1, MKN7, MKN28, MKN45, SNU1, SNU16, AGS, KatoIII, NCI-N87) were previously described [19]. Cells were maintained in RPMI 1640 medium (GIBCO, Grand Island, NY) supplemented with 10% fetal bovine serum (GIBCO, Grand Island, NY). Among them, AGS (poorly differentiated and p53-wide type), NCI-N87 (well differentiated and p53-mutation) and MKN45 (with xenograft formation ability) were representatively chosen for functional studies.

The primary paired samples from gastric cancer patients were randomly selected from Prince of Wales Hospital (Year 1999–2010). All participants provided written consent for the research experiment. Ethical approval was obtained from the Joint Chinese University of Hong Kong-New Territories East Cluster Clinical Research Ethics Committee (CREC Ref. No.2009.521).

### RNA extraction and quantitative real-time polymerase chain reaction (qRT-PCR)

The normal tissue corresponds to Human Stomach Total RNA commercially available from Ambion (AM7996, Grand Island, NY). A total of 67 paired (tumor and corresponding adjacent non-tumorous tissue) RNA samples were achieved from frozen tissues and 9 paired samples were got from micro-dissection of paraffin embedded tissues. RNA was extracted using TRIzol reagent (Invitrogen, Grand Island, NY). High Capacity cDNA Reverse Transcription Kits (Applied Biosystems, Grand Island, NY) were used for cDNA synthesis. qRT-PCR was used to quantitative differences in mRNA expression of AKT2 and primers were as following (sense-Exon 9: CAA AGA TGG CCA CAT CAA GA; anti-sense-Exon 10: GTC ATT GTC CTC CAG CAC CT). AKT1 expression were detected using the following primers (sense-Exon 9: GAG ATT GTG TCA GCC CTG GA; anti-sense-Exon 10: AGC CCG AAG TCT GTG ATC TT). The relative expression level was normalized by RPL29 (sense-Exon 3: GGA CCC CAA GTT CCT GAG G; anti-sense-Exon 4: GCA TTG TTG GCC TGC ATC TT) in gastric tissues and B2M (sense-Exon 1: ACT CTC TCT TTC TGG CCT GG; anti-sense-Exon 2: ATG TCG GAT GGA TGA AAC CC) in gastric cancer cell lines [20]. PCR was performed using SYBR Green PCR reagents (Applied Biosystems) according to the manufacturer’s instructions. The reactions were incubated in a 96-well plate at 95°C for 10 min, followed by 40 cycles of 95°C for 15 seconds and 60°C for 1 minute.

For miRNA expression detection, Taqman miRNA assays were used to quantify the expression levels of mature let-7b and let-7g (KIT, 002619 and 002282, Applied Biosystems). The relative expression level of microRNAs was normalized by RNU6B (KIT, 001093, Applied Biosystems). The reactions were performed in 7500 Fast Real-Time System (Applied Biosystems) and the reaction mix was incubated at 95°C for 30 seconds, followed by 40 cycles of 95°C for 8 seconds and 60°C for 30 seconds.

### Protein extraction and Western blot analysis

Protein was extracted from gastric cancer cell lines and paired primary tissues using RIPA lysis buffer with proteinase inhibitor. Protein concentration was measured by the method of Bradford (Bio-Rad, Hercules, CA) and 20 μg of protein mixed with 2 × SDS loading buffer was loaded per lane, separated by 12% SDS-polyacrylamide gel electrophoresis. Protein expression was detected using primary monoclonal anti-AKT2 antibody (1:1000 dilution, #3063, Cell Signaling, Danvers, MA), anti-phospho-AKT (S473) (1:1000 dilution, #9271, Cell Signaling) and anti-pS6 antibody (1:1000, #4858, Cell Signaling). The secondary antibodies were anti-Mouse IgG-HRP (1:30000, 00049039, Dako, Glostrup, Denmark) and anti-Rabbit IgG-HRP (1:10000, 00028856, Dako). The Western blot bands were quantified by ImageJ.

### miRNA/siRNA (small inference RNA) transfection and functional study

The miRNA precursors, let-7b (PM11050), let-7g (PM11758), scramble control (AM17110) were purchased from Applied Biosystems. And siAKT2 (SI00287672 and SI00287679) were obtained from Qiagen (Valencia, CA). All transfection were performed using Lipofectamine 2000 Transfection Reagent (Invitrogen) in a 20 nM concentration for 48 hours followed with functional study and RNA/protein analysis. Cell proliferation was assessed using CellTiter 96 Non-Radioactive Cell Proliferation Assay (Promega, Madison, WI) according to manufacturer’s instruction. For colony formation assays in monolayer cultures, the transfected cells were cultured in 6-well plates for 10 days. Cells were fixed with 70% ethanol for 15 minutes and stained with 2% crystal violet. The cell invasion assays using BD Biocoat Matrigel Invasion Chambers (BD Biosciences, Franklin Lakes, NJ) has been described previously by W. Kang [21]. The experiment was performed in triplicate and the mean including SDs was plotted.

### Rescue experiments

let-7b and let-7g precursors together with the negative control were transfected in AGS, NCI-N87 and MKN45 cells. And 24 hours after precursor transfection, AKT2 expression plasmid (from Addgene, Plasmid #16000) and empty plasmid (pcDNA3, Life Technologies, Grand Island, NY) were subsequently transfected with FuGENE HD Transfection Reagent (Roche, Nutley, NJ). After another 24 hours, cells were collected for functional study (MTT proliferation assays, monolayer colony formation assays and *in vivo* animal model). The experiments were repeated for 3 times and the representative data was shown.

### Luciferase assays

The annealed oligonucleotides containing the putative let-7b/g binding site was cloned into pMIR-REPORT vector (Ambion) via *HindIII* and *SpeI*. The sequences of oligonucleotides were (sense) AGA CAC TAG TTA GCA CTT CAC ACC CAT TGA and (antisense) GAG AAA GCT TCT AGA GAT TAC AGG CAT GAG CCA CT). Mutations located on the predicted seed binding region were introduced into the luciferase reporter vector using QuickChange® II Site-Directed Mutagenesis Kit according to the manufacturer’s instruction. The primer sequences for mutagenesis were 5′-GTG TGG GCA CAG GCC TGG GTT CGT GAT CTT TTT AGT GCC TCT C-3′ and 5′-GAG AGG CAC TAA AAA GAT CAC GAA CCC AGG CCT GTG CCC ACA C-3′. The firefly luciferase reporter construct and the control Renilla luciferase construct were co-transfected into NCI-N87 cells in 24-well plates. Cells were harvested after 36 hours for reporter activity analysis using Dual Luciferase Reporter assays (Promega, Madison, WI) as described previously [22].

### *In vivo* tumorigenicity study

Total three batches of animal models were used in this study. For let-7b/g or siAKT2 functional study, MKN45 cells were transfected with let-7b/g precursors or siAKT2 together with their control counterparts. The cells (5 × 10^6^ cells suspended in 0.1 ml PBS) were injected subcutaneously into the dorsal flank of eight 4-week-old male Balb/c nude mice. For the rescue *in vivo* study, MKN45 was transfected with let-7b/g for 24 hours following by AKT2 or empty vector transfection for another 24 hours. Then the cells were injected subcutaneously into the dorsal flank of eight Balb/c nude mice. Tumor diameter was measured and documented every 4 days until the tumor reached 10 mm in diameter. Tumor volume (mm^3^) was estimated by measuring the longest and shortest diameter of the tumor and calculated using the following formula: volume = (shortest diameter)^2^ × (longest diameter) × 0.5 [23]. All animal handling and experimental procedures were approved by Department of Health, Hong Kong (Reference No: 12–241 in DH/HA&P/8/2/1).

### Statistical analysis

The Student *T* test was employed to compare the functional effect between the target transfectants and the controls. Expression of let-7b/g and AKT2 (mRNA and protein) in primary cancerous tissues and their corresponding paired noncancerous tissues were compared by paired *T* test. Correlations between let-7b/g expression and clinicopathologic parameters were assessed by Pearson correlation analysis. The let-7b/g expression in gastric cancer cell lines was compared with it copy number change by non-parametric Spearman’s rho rank test. The Kaplan-Meier method was employed to estimate the survival rates for each variable. The equivalences of the survival curves were tested by log-rank statistics. All statistical analysis was performed by SPSS software (version 16.0; SPSS Inc). A two-tailed *P* value of less than 0.05 was considered statistically significant.

## Results

### The expression of let-7b/g is down-regulated in gastric cancer and correlates with poor survival

**Figure 1:**
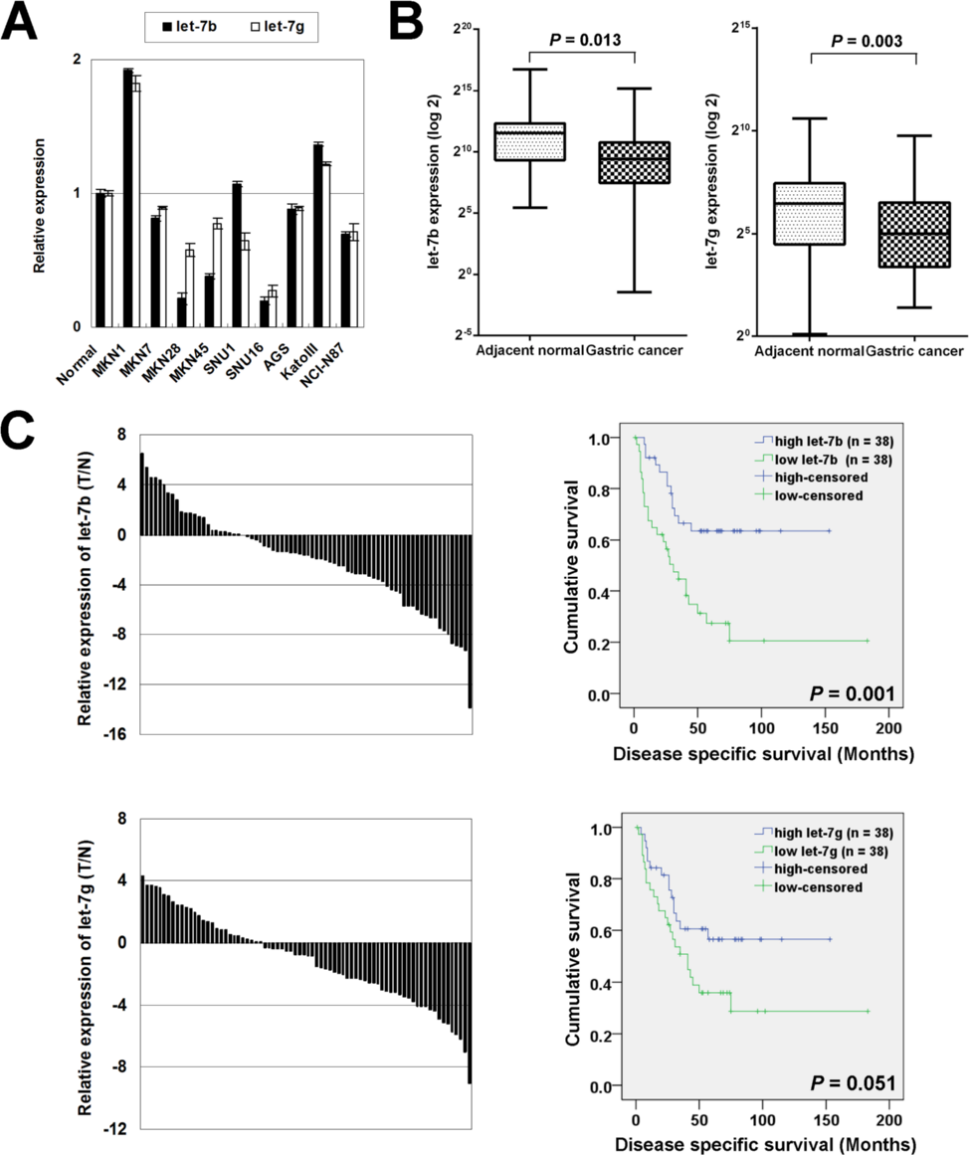
**let-7b/g is down-regulated in in gastric cancer and correlates with poor survival. (A)** The expression of let-7b/g in 9 gastric cancer cell lines compared with normal gastric epithelium tissue (AM7996). **(B)** let-7b/g showed a decreased expression in primary gastric tumors compared with paired adjacent non-tumours tissues (n = 76; let-7b, *P* = 0.013; let-7g, *P* = 0.003). **(C)** Downregulation of let-7b/g predicted poorer survival in clinical gastric cancer patients (let-7b, *P* = 0.001; let-7g, *P* = 0.051).

The expression of let-7b/g was measured in gastric cancer cell lines and normal stomach epithelium tissue by miRNA qRT-PCR. let-7b and let-7g were observed down-regulated in 6 and 7 gastric cancer cell lines respectively compared with normal gastric epithelial sample (Figure 1A). In a total of 76 paired primary RNA samples, the expression of let-7b and let-7g showed a decreased level in tumors compared with the corresponding non-tumorous gastric mucosal samples (let-7b, *P* = 0.013; let-7g, *P* = 0.003; Figure 1B).

In 76 paired primary samples, let-7b was found down-regulated in 53 (69.7%) tumor tissues which normalized by its expression in adjacent normal gastric tissue. Then two groups were stratified according to the median expression of let-7b and each group had the same 38 cases. The low-expression of let-7b group showed a poor survival compared with high-expression group (*P* = 0.001, up panel of Figure 1C). Meanwhile let-7g showed decreased expression in 48 (63.2%) tumor tissues when compared with adjacent normal tissues and its downregulation also predicted poorer prognosis in gastric cancer with a marginal significant *P*-value (*P* = 0.051, lower panel of Figure 1C). We further investigated the clinicopathologic correlation of let-7b and let-7g. Additional file 2: Table S2 summarized the correlation of let-7b/g with other clinicopathologic parameters in gastric cancer patients. The decreased expression of let-7b was correlated with lymph node metastasis (*P* = 0.021). The let-7g downregulation was also associated with N-stage (*P* = 0.044) and lymph node metastasis (*P* = 0.009), suggesting that these two miRNAs might be involved in gastric cancer cell metastasis.

**Figure 2:**
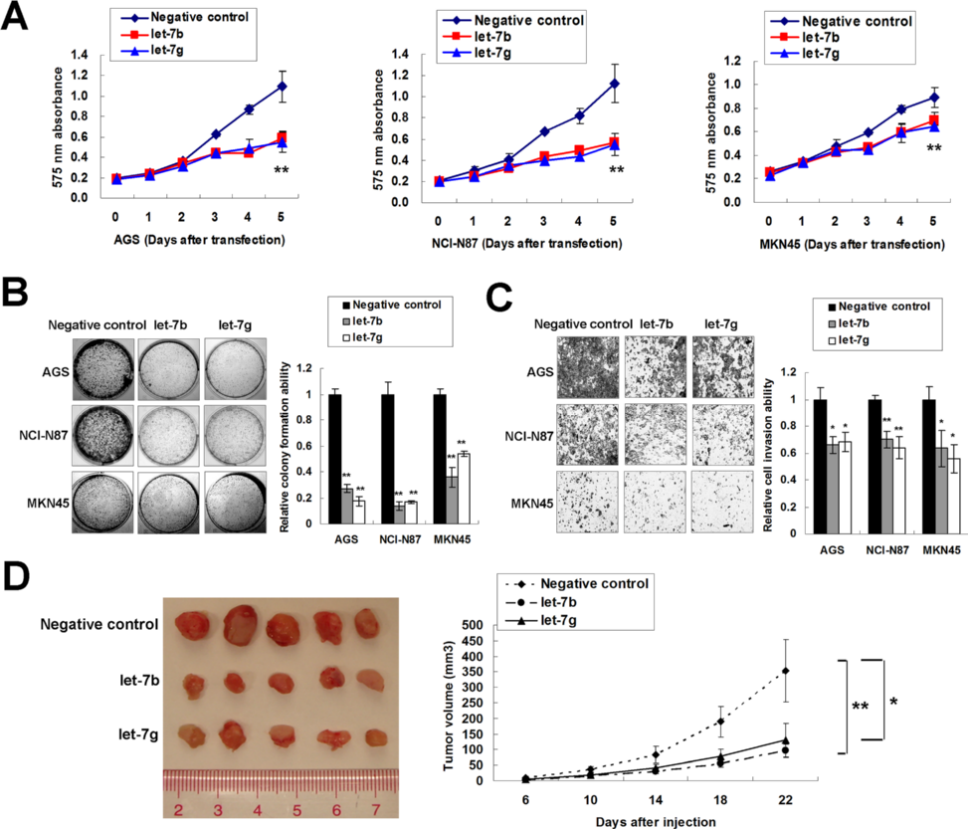
**Ectopic expression of let-7b/g exerts tumor suppresser function in gastric cancer cells. (A)** 5-day MTT proliferation results of let-7b/g in AGS, NCI-N87 and MKN45 cells (**, *P* < 0.001). **(B)** let-7b/g decreased monolayer colony formation in AGS, NCI-N87 and MKN45 cells (**, *P* < 0.001). The experiment was performed in triplicate wells to get standard deviations (SDs). **(C)** let-7b/g inhibited gastric cancer cell invasion (*, *P* < 0.05; **, *P* < 0.001). Three random vision fields were selected for invaded cell counting to get SDs. **(D)** let-7b/g-MKN45 formed smaller xenografts than the negative control group (*, *P* < 0.05; **, *P* < 0.001).

### let-7b and let-7g exert tumor suppressor function in gastric cancer cells

To investigate the functional role of let-7b/g, the precursors of these two miRNAs were transfected into AGS, NCI-N87 and MKN45 cells. let-7b/g suppressed the growth of gastric cancer cells AGS, NCI-N87 and MKN45 as demonstrated by a 5-day MTT assay (*P* < 0.001, Figure 2A). The tumor suppressor function of let-7b/g was further validated by monolayer colony formation. A significant reduction of colony number was observed in let-7b/g transfectants compared with scramble miRNA groups (*P* < 0.001, Figure 2B). Ectopic expression of let-7b or let-7g was further revealed to inhibit the invasive capacity of gastric cancer cells as demonstrated by Matrigel invasion assays (*P* < 0.05, Figure 2C). The MKN45 cells with let-7b/g transfection were injected subcutaneously into the dorsal flank of nude mice. let-7b/g transfectants formed smaller xenografts than those scramble transfectants after 22 days (let-7b, *P* < 0.001; let-7g, *P* < 0.05; Figure 2D).

**Figure 3:**
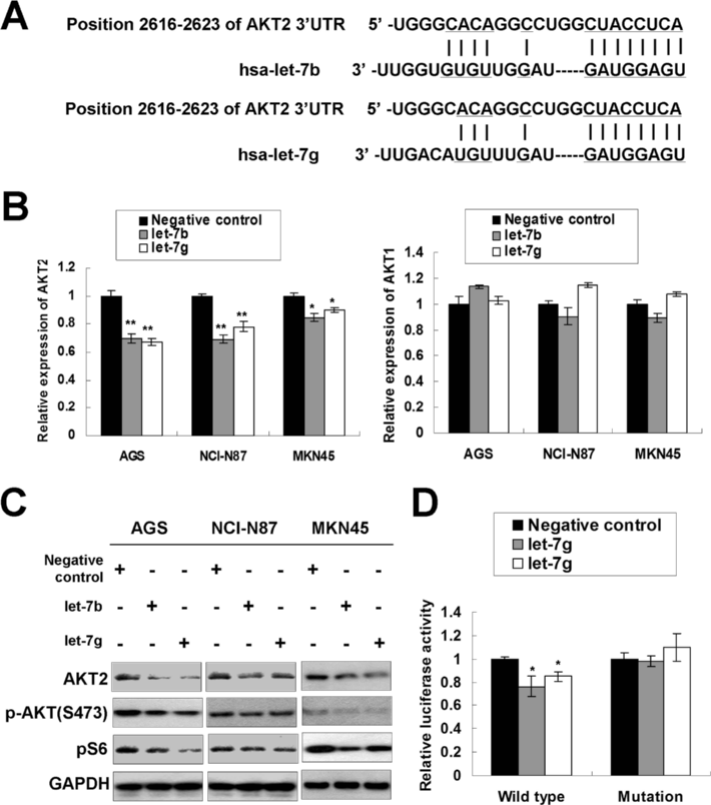
**let-7b/g targets AKT2 in gastric cancer. (A)** The binding sites of let-7b/g on the AKT2 3′UTR as predicted by TargetScan (www.targetscan.org). **(B)** AKT2 mRNA expression was down-regulated upon ectopic let-7b/g expression (*, *P* < 0.05; **, *P* < 0.001). **(C)** Western blot analysis of AKT2, p-AKT (S473) and pS6 upon let-7b/g ectopic expression in AGS, NCI-N87 and MKN45 cells. **(D)** Relative luciferase activity in the constructs containing wild-type binding site or mutated binding site of AKT2 3′UTR after let-7b/g transfection (Wild type, the construct containing the complementary sequence of seed region; Mutation, the binding site was mutated; *, *P* < 0.05).

### let-7b and let-7g decrease AKT2 expression by direct binding with its 3′UTR

AKT2 was predicted to be a putative target of let-7b/g by TargetScan (www.targetscan.org). The putative binding sites of let-7b/g within the 3′UTR of AKT2 were shown in Figure 3A. We transfected let-7b/g into gastric cancer cells and decreased AKT2 mRNA expression level was observed in AGS, NCI-N87 and MKN45 cells (*P* < 0.05; left bar chart of Figure 3B). However, ectopic expression of let-7b/g did not change the AKT1 mRNA expression (right bar chart of Figure 3B), indicating let-7b/g only exerts its inhibitory effect on AKT2 but not on AKT1. AKT2 protein showed a decrease expression after ectopic let-7b/g expression (Figure 3C), suggesting that let-7b/g triggered a silencing effect on the endogenous AKT2 expression. Meanwhile, the phosphorylated AKT (S473) and its direct downstream effector of AKT-mTOR pathway, pS6, showed decreased expression upon let-7b/g overexpression. A luciferase reporter assay was performed to test whether AKT2 is a direct target of let-7b/g. As shown in Figure 3D, let-7b/g decreased the relative luciferase activity of constructs encompassing AKT2 3′UTR binding sites in NCI-N87 cells (*P* < 0.05). Meanwhile, let-7b/g did not suppress the luciferase activity in the constructs containing the mutated binding site. The results in this part revealed that let-7b/g specifically recognized the binding sites in AKT2 3′UTR and directly repressed AKT2 expression through mRNA degradation and protein synthesis inhibition.

**Figure 4:**
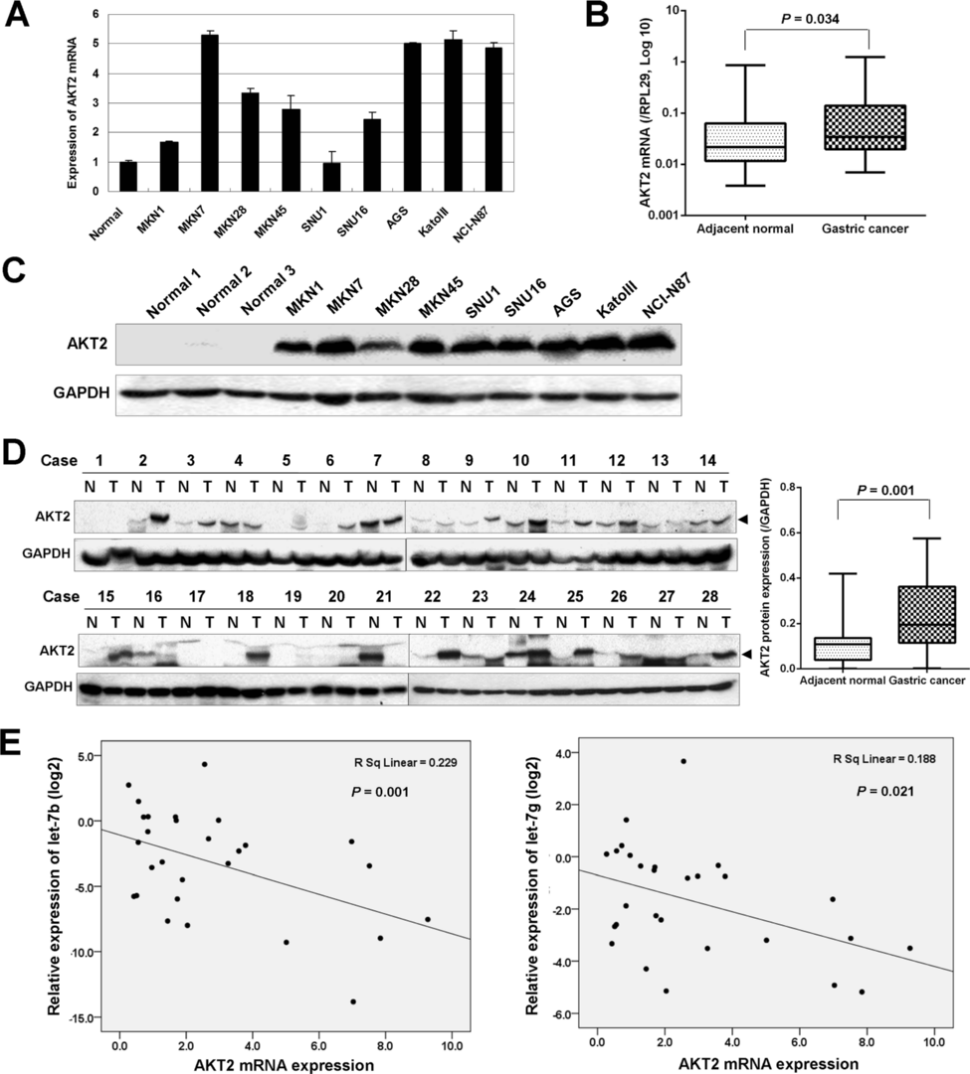
**AKT2 is over-expressed in gastric cancer. (A)** The expression of AKT2 mRNA in 9 gastric cancer cell lines compared with normal gastric tissue. **(B)** AKT2 mRNA showed increased expression in gastric tumor tissues compared with adjacent non-cancerous tissues (n = 28, *P* = 0.034). **(C)** Western blot analysis of AKT2 in 9 gastric cancer cell lines and 3 normal gastric tissues. **(D)** The protein expression of AKT2 in 28 paired primary tumors (N, paired non-tumorous tissue; T, tumor tissue). The AKT2 protein expression in gastric cancer tissues was up-regulated compared with adjacent non-tumorous tissues by quantification (*P* = 0.001). **(E)** AKT2 mRNA expression showed negative correlation with let-7b/g expression in primary tumors (n = 28; let-7b, *P* = 0.001; let-7g, *P* = 0.021).

### AKT2 is up-regulated in gastric cancer and shows negative correlation with let-7b/g

Expression of AKT2 mRNA was up-regulated in 8 gastric cancer cell lines except SNU1 compared with normal gastric mucosal sample (Figure 4A). In 28 pairs of primary gastric cancers, AKT2 mRNA expression showed upregulation in 19 (67.9%) tumor samples by normalization with its expression in adjacent non-tumorous tissues. The expression of AKT2 in the cancer tissues showed higher expression than it expression in adjacent normal gastric tissues (*P* = 0.034, Figure 4B). Strong AKT2 protein expression can be strongly detected in all 9 gastric cancer cell lines but only one normal gastric mucosal tissue showed week AKT2 protein expression (Figure 4C). In 28 paired primary protein samples, AKT2 protein expression showed up-regulated in the cancer tissues compared with adjacent non-tumorous tissues after quantification (*P* = 0.001, Figure 4D). We then examined the AKT2 mRNA and let-7b/g expression in 28 paired primary gastric cancer samples. The AKT2 mRNA expression was negatively correlated with let-7b/g expression (let-7b, *P* = 0.001; let-7g, *P* = 0.021; Figure 4E), suggesting let-7b/g downregulation might be, at least partly, responsible for the aberrant activation of AKT2 in gastric cancers.

**Figure 5:**
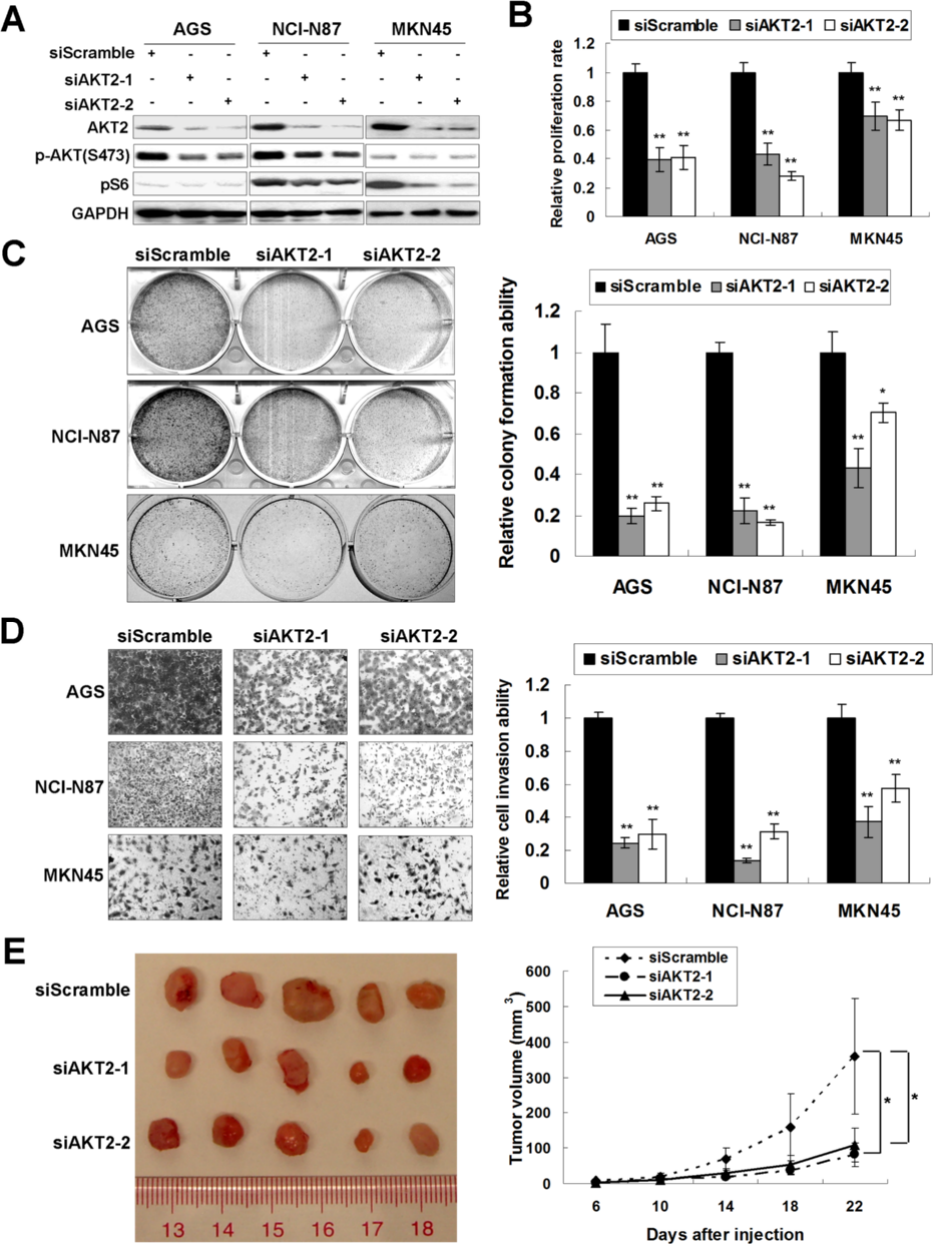
**AKT2 knockdown phenocopies the tumor-suppressive effect of let-7b/g in gastric cancer cells. (A)** Western blot of AKT2, p-AKT (S473) and pS6 after siAKT2 transfection in AGS, NCI-N87 and MKN45 cells. **(B)** AKT2 knockdown suppressed cell proliferation (**, *P* < 0.001). 6 wells were measured for each group to get SDs. **(C)** AKT2 knockdown decreased monolayer colony formation in AGS, NCI-N87 and MKN45 cells (**, *P* < 0.001). The experiments was repeated in 3 wells to get SDs. **(D)** siAKT2 inhibited cell invasion of gastric cancer cells (**, *P* < 0.001). The invaded cells in 3 random vision fields were counted for SDs achieving. **(E)** siAKT2 suppressed the formation of xenografts in nude mice compared with siScramble group (*, *P* < 0.05).

### siRNA-mediated knockdown of AKT2 phenocopies the tumor-suppressive effect of let-7b/g

We have demonstrated that let-7b/g targeted AKT2 and exerted tumor suppressor function in gastric cancer. siRNA-mediated AKT2 knockdown was therefore performed to investigate if AKT2 downregulation phenocopied the tumor-suppressive effect of let-7b/g. We transfected siAKT2 into gastric cancer cell lines AGS, NCI-N87 and MKN45. Successful AKT2 knockdown was confirmed by Western blot analysis. p-AKT (S473) and pS6 protein showed decreased expression upon siAKT2 transfection (Figure 5A). AKT2 knockdown suppressed AGS, NCI-N87 and MKN45 cell proliferation in a 5-day MTT assays (*P* < 0.001, Figure 5B). AKT2 knockdown also decreased monolayer colony formation in gastric cancer cells to a significant level (Figure 5C). Moreover, siAKT2 inhibited cell invasion of gastric cancer cells (*P* < 0.001, Figure 5D). The effect of siAKT2 on tumor growth *in vivo* was also investigated. The siAKT2-MKN45 and vector control cells were injected into nude mice subcutaneously. The tumor growth in siAKT2 groups was significantly decreased compared with the vector control after 22 days (*P* < 0.05, Figure 5E).

### AKT2 re-expression abrogated the growth-inhibitory effect of let-7b/g

**Figure 6:**
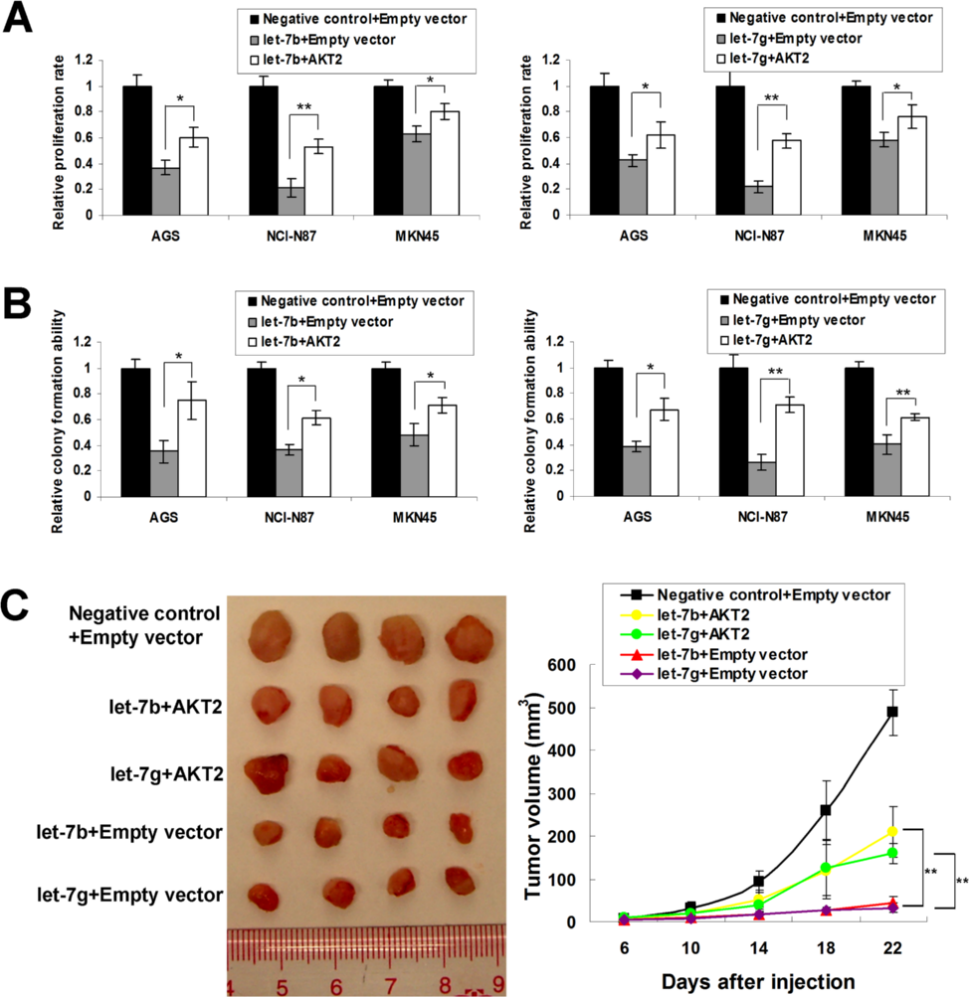
**AKT2 re-expression rescues the inhibitory effect of let-7b/g. (A)** AKT2 re-expression partly abolished the growth-inhibitory effect of let-7b/g in a 5-day MTT assays (*, *P* < 0.05; **, *P* < 0.001). The SDs were achieved by the 575 nm absorbance readings in 6 wells of each item. **(B)** AKT2 abrogated the suppressed monolayer colony formation by let-b/g (*, *P* < 0.05; **, *P* < 0.001). The experiments was performed in triplicate wells to get SDs. **(C)** AKT2 re-expression promoted *in vivo* tumor growth (**, *P* < 0.001).

Since AKT2 is a predicted target of let-7b/g, we further investigated if AKT2 re-expression diminished the suppressive phenotype change caused by let-7b and let-7g. Interestingly, we found that the growth inhibitory effect of let-7b/g were partially abrogated by AKT2 re-expression (MTT proliferation assays, Figure 6A; monolayer colony formation assays, Figure 6B), suggesting AKT2 was functionally involved in let-7b/g-inducing suppression of gastric cancer cell growth. *In vivo* experiments yielded similar results. Cells co-transfected with let-7b/g and AKT2 formed bigger xenografts than those with let-7b/g alone (*P* < 0.001, Figure 6C).

## Discussion

let-7 family, which has 10 mature subtypes identified (from 7a to 7i, miR-98 and miR-202), is closely associated with normal development and human cancers [24]. LIN28 and LIN28B have been found to act as post-transcriptional repressors of let-7 biogenesis by binding with the loop portion of the pri-let-7 hairpin and the stem of pre-let-7 to inhibit its binding with Drosha or Dicer [25]. Genomic DNA copy number alterations [26] and DNA methylation [27] are also responsible for the decreased expression of let-7 family in cancers. In 9 gastric cancer cells, we compared let-7b (in 22q13) and let-7g (in 3p21) expression with the DNA copy number change of their loci and found although the *P*-value is not significant due the limited number of cell lines, let-7b/g expression showed a trend of positive correlation with their array-CGH results (let-7b, *P* = 0.205; let-7g, *P* = 0.088; Additional file 3: Figure S1).

The let-7 miRNA family is involved in the proliferation, apoptosis and invasion of cancer cells. A number of studies have reported that let-7 is down-regulated and acts as a tumor suppressor in kinds of cancer types, including non-small cell lung carcinoma [28], breast cancer [29], prostate cancer [30], nasopharyngeal carcinoma [31], hepatocellular carcinoma [32] and esophageal squamous cell carcinoma. Several oncogenes and signaling pathways, such as RAS oncogene, c-Myc [33], HMGA2 [15] and JAK-STAT3 pathway [24], are the main targets of let-7 in carcinogenesis. In addition, SNP rs61764370 in KRAS 3′UTR (T. > G) promotes cell proliferation through downregulation of let-7a/b/c [34]. In current study, we not only confirmed the tumor suppressor function of let-7b/g but also revealed a novel functional target of let-7 family, AKT2, together with the dysregulated signaling pathway, AKT2-mTOR-pS6. Ectopic expression of let-7b/g down-regulated AKT2 and its downstream effector pS6. In addition, AKT2 re-expression alleviated the suppressive phenotypes of let-7b/g, thus establishing their functional interaction. More importantly in primary samples, AKT2 mRNA expression showed a negative correlation with let-7b/g expression, suggesting that let-7b/g exerts its tumor suppressor function in gastric carcinogenesis at least by partly down-regulating AKT2. All these findings in this part revealed the critical role of let-7b/g in molecular gastric carcinogenesis.

AKT2, a member of Protein Kinase B family, is an important signaling molecule in the insulin signaling pathway. It has been identified as an oncogene and amplified in human ovarian carcinomas [35], pancreatic carcinomas [36] and pancreatic ductal adenocarcinomas [37]. AKT2 is a link between the metabolic action of insulin signal transduction and tumorigenesis in liver malignancies [38]. As a major downstream effector of PI3K/AKT survival pathway, AKT2 expression markedly increased the incidence of pulmonary metastases in breast tumor model [39]. AKT2 knockdown by RNA interference suppresses cell proliferation and induces apoptosis even increases chemosensitivity in lung adenocarcinoma [40], malignant gliomas [41,42], pancreatic cancer [43] and ovarian cancer [44]. We showed that AKT2 was upregulated in gastric cancer and its knockdown suppressed gastric cancer cell proliferation, reduced monolayer colony formation and inhibited xenograft formation in vivo, resembling the tumor-suppressive effects of let-7b/g in gastric cancer cells.

In conclusion, our results underscore let-7b/g as important tumor-suppressive miRNAs in gastric cancer cells by directly targeting AKT2. All these findings support that the frequently down-regulated let-7b/g contributes to activation of AKT2 and gastric carcinogenesis which might has therapeutic potential in gastric cancer.

## Additional files

Additional file 1: Table S1. Relative expression of let-7 family in gastric cancer cell lines compared with normal gastric tissue (from microRNA expression microarray data, log2 ratio). Additional file 2: Table S2. Correlation of let-7b and let-7g expression with clinicopathologic features (significant *P*-value in bold and Italic format). Additional file 3: Figure S1. The correlation of let-7b/g expression with DNA copy number change of its loci in gastric cancer cell lines (n = 9). (A) The correlation of let-7b expression with copy number change of 22q13 (*P* = 0.205). (B) let-7g expression showed a trend of positive correlation with its copy number change in 3p21 (*P* = 0.088).
