# Supplementary material for: let-7b/g silencing activates AKT signaling to promote gastric carcinogenesis
Source: J Transl Med. 2014 Oct 5;12:281. doi: 10.1186/s12967-014-0281-3 (PMC4196013; doi:10.1186/s12967-014-0281-3)
Supplement: Additional file 1: Table S1. — Relative expression of let-7 family in gastric cancer cell lines compared with normal gastric tissue (from microRNA expression microarray data, log2 ratio). [file 12967_2014_281_MOESM1_ESM.doc]

**Table S1** **Relative expression of let-7 family in gastric cancer cell lines compared with normal gastric tissue (from microRNA expression microarray data, log2 ratio).**

| let-7 family | AGS | MKN1 | MKN28 | MKN45 | MKN7 | SNU1 | SNU16 | Normal tissue |
| --- | --- | --- | --- | --- | --- | --- | --- | --- |
| hsa-let-7a | -4,734 | -4,461 | -4,449 | -4,607 | -4,637 | -3,912 | -3,433 | 0 |
| hsa-let-7b | -4,346 | -4,284 | -4,739 | -4,226 | -4,665 | -3,084 | -3,076 | 0 |
| hsa-let-7c | -840 | -794 | -879 | -828 | -870 | -276 | -708 | 0 |
| hsa-let-7d | -780 | -766 | -572 | -845 | -881 | -729 | -437 | 0 |
| hsa-let-7e | -372 | -747 | -455 | -898 | -750 | -741 | -926 | 0 |
| hsa-let-7f | -1,974 | -1,884 | -1,748 | -1,906 | -1,988 | -1,749 | -1,100 | 0 |
| hsa-let-7g | -860 | -882 | -857 | -755 | -908 | -836 | -579 | 0 |
| hsa-let-7i | -292 | -243 | -268 | -304 | -352 | -278 | -246 | 0 |
