# Supplementary material for: let-7b/g silencing activates AKT signaling to promote gastric carcinogenesis
Source: J Transl Med. 2014 Oct 5;12:281. doi: 10.1186/s12967-014-0281-3 (PMC4196013; doi:10.1186/s12967-014-0281-3)
Supplement: Additional file 2: Table S2. — Correlation of let-7b and let-7g expression with clinicopathologic features (significant P-value in bold and Italic format). [file 12967_2014_281_MOESM2_ESM.doc]

**Table S2 Correlation of let-7b and let-7g expression with clinicopathologic features (significant *P*-value in bold and Italic format)**.

|  |  | let-7b expression | | | let-7g expression | | |
| --- | --- | --- | --- | --- | --- | --- | --- |
|  |  | high (%) | low (%) | *P*-value | high (%) | low (%) | *P*-value |
| Sex | M | 14 (40.0) | 21 (60.0) | 0.167 | 17 (48.6) | 18 (51.4) | 1.000 |
|  | F | 24 (58.5) | 17 (41.5) |  | 21 (51.2) | 20 (48.8) |  |
| Age | <=60 | 13 (54.2) | 11 (45.8) | 0.805 | 14 (58.3) | 10 (41.7) | 0.460 |
|  | >60 | 25 (48.1) | 27 (51.9) |  | 22 (44.0) | 28 (56.0) |  |
| Type | Intestinal | 18 (56.3) | 14 (43.7) | 0.486 | 16 (50.0) | 16 (50.0) | 1.000 |
|  | Diffuse | 20 (45.5) | 24 (54.5) |  | 22 (50.0) | 22 (50.0) |  |
| Grade | 1 | 1 (100) | 0 (0) | 0.055 | 1 (100.0) | 0 (0.0) | 0.055 |
|  | 2 | 29 (56.9) | 22 (43.1) |  | 29 (56.9) | 22 (43.1) |  |
|  | 3 | 6 (28.6) | 15 (71.4) |  | 6 (28.6) | 15 (71.4) |  |
| Stage (T) | 1 | 9 (81.8) | 2 (18.2) | 0.100 | 8 (72.7) | 3 (27.3) | 0.360 |
|  | 2 | 13 (52.0) | 12 (48.0) |  | 13 (52.0) | 12 (48.0) |  |
|  | 3 | 11 (42.3) | 15 (57.7) |  | 11 (42.3) | 15 (57.7) |  |
|  | 4 | 5 (35.7) | 9 (64.3) |  | 6 (42.9) | 8 (57.1) |  |
| Stage (N) | 0 | 15 (71.4) | 6 (28.6) | 0.059 | 16 (76.2) | 5 (23.8) | ***0.044*** |
|  | 1 | 9 (40.9) | 13 (59.1) |  | 9 (40.9) | 13 (59.1) |  |
|  | 2 | 8 (57.1) | 6 (42.9) |  | 6 (42.9) | 8 (57.1) |  |
|  | 3 | 6 (31.6) | 13 (68.4) |  | 7 (36.8) | 12 (63.2) |  |
| Stage (M) | 0 | 31 (50.8) | 30 (49.2) | 1.000 | 31 (50.8) | 30 (49.2) | 1.000 |
|  | 1 | 7 (46.7) | 8 (53.3) |  | 7(46.7) | 8 (53.3) |  |
| Lymph Node | 0 | 16 (72.7) | 6 (27.3) | ***0.021*** | 16 (72.7) | 6 (27.3) | ***0.009*** |
|  | 1 | 23 (41.8) | 32 (58.2) |  | 22 (40.0) | 33 (60.0) |  |
| *H. pylori* | Absence | 28 (49.1) | 29 (50.9) | 0.566 | 30 (52.6) | 27 (47.4) | 1.000 |
|  | Presence | 9 (60.0) | 6 (40.0) |  | 8 (53.3) | 7 (46.7) |  |
