# Supplementary figures and images for: let-7b/g silencing activates AKT signaling to promote gastric carcinogenesis
Source: J Transl Med. 2014 Oct 5;12:281. doi: 10.1186/s12967-014-0281-3 (PMC4196013; doi:10.1186/s12967-014-0281-3)

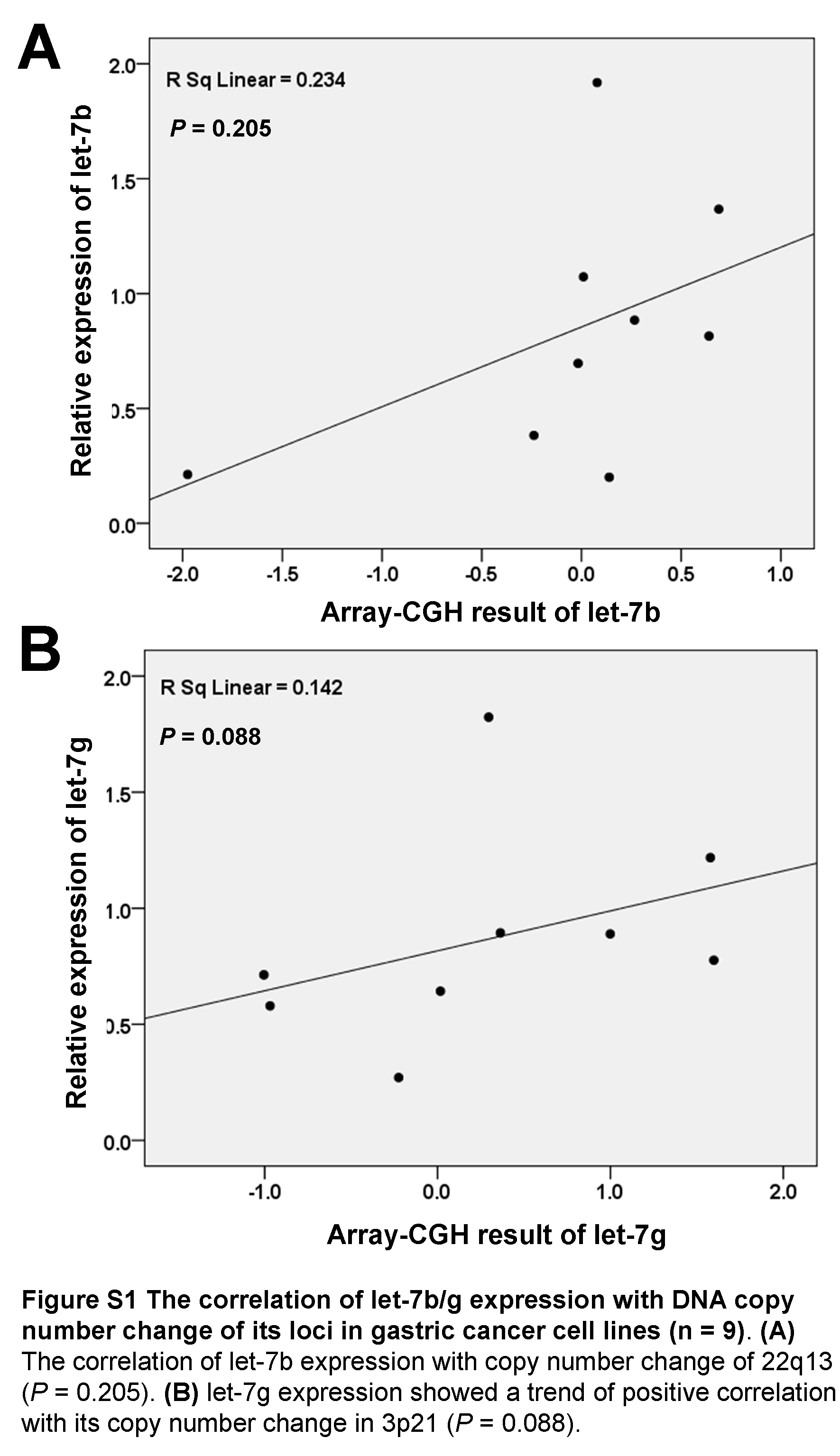

Supplement: Additional file 3: Figure S1. — The correlation of let-7b/g expression with DNA copy number change of its loci in gastric cancer cell lines (n = 9). (A) The correlation of let-7b expression with copy number change of 22q13 (P = 0.205). (B) let-7g expression showed a trend of positive correlation with its copy number change in 3p21 (P = 0.088). [file 12967_2014_281_MOESM3_ESM.tif]
